# Correction: Isatin-based benzyloxybenzene derivatives as monoamine oxidase inhibitors with neuroprotective effect targeting neurodegenerative disease treatment

**DOI:** 10.1039/d3ra90123h

**Published:** 2024-01-02

**Authors:** Feba Benny, Jong Min Oh, Sunil Kumar, Mohamed A. Abdelgawad, Mohammed M. Ghoneim, Mohamed Sadek Abdel-Bakky, Neelima Kukerti, Jobin Jose, Hoon Kim, Bijo Mathew

**Affiliations:** a Department of Pharmaceutical Chemistry, Amrita School of Pharmacy, Amrita Vishwa Vidyapeetham, AIMS Health Sciences Campus Kochi 682041 India febacbenny@gmail.com solankimedchem@gmail.com bijovilaventgu@gmail.com bijomathew@aims.amrita.edu; b Department of Pharmacy, Research Institute of Life Pharmaceutical Sciences, Sunchon National University Suncheon 57922 Republic of Korea ddazzo005@naver.com hoon@sunchon.ac.kr; c Department of Pharmaceutical Chemistry, College of Pharmacy, Jouf University Sakaka 72341 Saudi Arabia mhmdgwd@ju.edu.sa; d Department of Pharmacy Practice, College of Pharmacy, AlMaarefa University Ad Diriyah 13713 Saudi Arabia mghoneim@mcst.edu.sa; e Department of Pharmacology and Toxicology, College of Pharmacy, Qassim University Buraydah 51452 Saudi Arabia abdelbakkym@yahoo.com; f School of Pharmacy, Graphic Era Hill University Dehradun Uttarakhand 248002 India nkukreti@gehu.ac.in; g Department of Pharmaceutics, NGSM Institute of Pharmaceutical Science, NITTE University Mangalore Karnataka 575018 India jjmattam07@gmail.com

## Abstract

Correction for ‘Isatin-based benzyloxybenzene derivatives as monoamine oxidase inhibitors with neuroprotective effect targeting neurodegenerative disease treatment’ by Feba Benny *et al.*, *RSC Adv.*, 2023, **13**, 35240–35250, https://doi.org/10.1039/D3RA07035B.

The authors regret that the title shown in the original article was incorrect. The correct title is as shown here.

The Royal Society of Chemistry apologises for these errors and any consequent inconvenience to authors and readers.

## Supplementary Material

